# The Role of Gender in the Transition to Driving Cessation in Persons With Dementia

**DOI:** 10.1093/geroni/igaa057.969

**Published:** 2020-12-16

**Authors:** Stephanie Yamin, Roxana Manoiu, Gary Naglie, Sarah Sanford, Elaine Stasiulis, Brenda Vrkljan, Mark Rapoport

**Affiliations:** 1 Saint Paul University, Ottawa, Ontario, Canada; 2 Saint-Paul University, Saint-Paul University, Ontario, Canada; 3 University of Toronto, Toronto, Ontario, Canada; 4 Baycrest Health Sciences, Toronto, Ontario, Canada; 5 Baycrest Rotman Research Institute, Toronto, Canada; 6 McMaster University, Hamilton, Ontario, Canada; 7 Sunnybrook Health Sciences Centre, Sunnybrook Health Sciences Centre, Ontario, Canada

## Abstract

Driving often provides a sense of independence, quality of life and emotional wellbeing. For older adults living with dementia, driving cessation eventually becomes inevitable. Driving cessation has been shown to negatively impact older adults’ mobility and, consequently, quality of life. Caregivers of persons with dementia (PWD) who have ceased driving are also impacted as they often become responsible for meeting the mobility needs of PWD and they provide emotional support in respect to this significant life transition. To date, there is little information on the role of gender in the transition to driving cessation in PWD. The purpose of this study was to examine the role that gender plays among drivers and ex-drivers with dementia from the perspectives of PWD, their caregivers, and healthcare practitioners. Secondary thematic analyses were conducted from a pre-existing sample of persons with dementia (N=10), family caregivers (N=13), and healthcare practitioners (N=6) who participated in interviews and focus groups about their experiences around driving cessation in the context of dementia. Data analyses involved an inductive thematic technique that allowed for generating themes. The main themes identified gender differences as a significant factor in: (1) difficulty accepting driving cessation (2) driving as it is tied to identity, (3) emotional responses to driving cessation, (4) driving as part of the caregiving role. The findings suggest that there is a need for tailored interventions for men and women who lose their ability to drive, in addressing their unique emotional responses and in supporting them through this important life transition.

